# JNK/SAPK Signaling Is Essential for Efficient Reprogramming of Human Fibroblasts to Induced Pluripotent Stem Cells

**DOI:** 10.1002/stem.2327

**Published:** 2016-03-04

**Authors:** Irina Neganova, Evgenija Shmeleva, Jennifer Munkley, Valeria Chichagova, George Anyfantis, Rhys Anderson, Joao Passos, David J. Elliott, Lyle Armstrong, Majlinda Lako

**Affiliations:** ^1^ Institute of Genetic MedicineInternational Centre for Life and Centre for Integrated Systems Biology of Ageing and Nutrition; ^2^ Institute for Ageing and HealthNewcastle University

**Keywords:** JNKs, SAPK, MKK4, MKK7, hiPSC, hESC

## Abstract

Reprogramming of somatic cells to the phenotypic state termed “induced pluripotency” is thought to occur through three consecutive stages: initiation, maturation, and stabilisation. The initiation phase is stochastic but nevertheless very important as it sets the gene expression pattern that permits completion of reprogramming; hence a better understanding of this phase and how this is regulated may provide the molecular cues for improving the reprogramming process. c‐Jun N‐terminal kinase (JNK)/stress‐activated protein kinase (SAPKs) are stress activated MAPK kinases that play an essential role in several processes known to be important for successful completion of the initiation phase such as cellular proliferation, mesenchymal to epithelial transition (MET) and cell cycle regulation. In view of this, we postulated that manipulation of this pathway would have significant impacts on reprogramming of human fibroblasts to induced pluripotent stem cells. Accordingly, we found that key components of the JNK/SAPK signaling pathway increase expression as early as day 3 of the reprogramming process and continue to rise in reprogrammed cells throughout the initiation and maturation stages. Using both chemical inhibitors and RNA interference of *MKK4, MKK7* and *JNK1*, we tested the role of JNK/SAPK signaling during the initiation stage of neonatal and adult fibroblast reprogramming. These resulted in complete abrogation of fully reprogrammed colonies and the emergence of partially reprogrammed colonies which disaggregated and were lost from culture during the maturation stage. Inhibition of JNK/SAPK signaling resulted in reduced cell proliferation, disruption of MET and loss of the pluripotent phenotype, which either singly or in combination prevented establishment of pluripotent colonies. Together these data provide new evidence for an indispensable role for JNK/SAPK signaling to overcome the well‐established molecular barriers in human somatic cell induced reprogramming. Stem Cells *2016;34:1198–1212*

**Significance Statement**

Our research group has a long standing interest in understanding the molecular mechanisms underpinning the induction of pluripotency which may be essential for enhancing the reprogramming process. In this manuscript, we have focused our attention on the c‐Jun N‐terminal kinase (JNK) signaling, a pathway which has been extensively studied in somatic and cancer cells, but it has been relatively unexplored in human pluripotent stem cells. Through a combination of techniques, we have been able to show that JNK/SAPK signaling is indispensable for overcoming several well described molecular barriers occurring in the initial stage of reprogramming, thus providing for the first time clear insights on the role of this pathway on human somatic cell induced reprogramming.

## Introduction

Since 2007, it has been possible to reprogram human somatic cells back to an embryonic stem cell (ESC) like stage via introduction of four transcription factors, namely OCT3/4, SOX2, KLF4, and c‐MYC (referred as OSKM; 1). The reprogrammed cells termed human induced pluripotent stem cells (hiPSCs), akin to human embryonic stem cells (hESCs) are characterized by the ability to proliferate indefinitely and have the potential to give rise to all cell types found in the adult organism 2. The aforementioned pluripotency factors have also been implicated in the initiation of tumorigenesis in various tissues 3, 4, 5 and are considered as potent oncogenes. The risk associated with the introduction of these oncogenes in normal human somatic cells is the likely activation of anti‐oncogenic pathways through a process named oncogenic stress which can often result in cell cycle arrest as a means of protection from tumorigenesis 6. Activation of cell cycle arrest, p53 activation and reduced cellular proliferation have been described as molecular barriers to efficient reprogramming 7, 8, 9. In view of these findings, we hypothesised that transduction of dermal skin (Fibroblasts: Lonza Group Ltd, Basel, Switzerland; Lonza:http://www.lonza.com/) with OSKM would activate oncogenic stress and induce growth arrest thus providing an additional barrier to reprogramming. Hence inhibition of key sensors of this pathway could provide useful targets for removing this barrier and increasing its efficiency.

Stress activated MAP kinase signaling pathways are important mediators of cellular responses to intra‐ and extracellular signals such as growth factors, hormones, and environmental stresses. These pathways consist of triple kinase cascades comprising the MAP kinases which are phosphorylated and activated by MAP kinase kinases (MKKs) and then further phosphorylated and activated by MAP kinase kinase kinases (MKKKs) 10. In mammals, three distinct MAP kinase pathways have been identified resulting in activation of ERK, c‐Jun N‐terminal kinase (JNK) and p38 10. One of the key sensors of oncogenic stress is the JNK signaling pathway which responds by phosphorylating and stabilising p53 via its downstream mediator MKK7 11. The JNK pathway, also named stress‐activated protein kinase pathway (SAPK) is essential for providing a cellular response to extracellular changes such as ultraviolet and reactive oxygen species induced damage, mechanical stress and osmolarity changes. For the JNK/SAPK pathway 14 different MKKs have been described to date 12. MKK7 exclusively activates JNKs and MKK4 is unique in its ability to phosphorylate and activate two MAP kinase groups: JNKs and p38. Both MKK4 and MKK7 are responsible for phosphorylation of JNK/SAPKs at Tyrosine (Tyr) and Threonine (Thr) residues located at the activation loop. In murine embryonic stem cells (mESCs), MKK4 is shown to phosphorylate JNK/SAPKs at Tyr 185 residue, while MKK7 phosphorylates the Thr 183 residue, and together they cause dual phosphorylation of JNK/SAPKs, thus leading to its optimal activation 13.

Most of the data on JNK/SAPK signaling and activation of its targets has been obtained from work performed in somatic and cancer cells 14; however in the last few years there have been a number of publications describing the role of this signaling pathway during embryonic development and in ESC function. Some of these have shown that MKK4 and MKK7 null mice die before E.12.5, highlighting the necessity of JNK/SAPK signaling pathway during embryonic development and suggesting that other pathways cannot substitute for MKK4 and MKK7 15, 16. Furthermore, JNK1 and JNK2 have been shown to play a negative role in the reprogramming of murine fibroblasts by suppressing Klf4 activity 17. In contrast, hESCs are characterized by high levels of JNK/SAPK activity which is important for maintenance of pluripotency; however a role for this signaling pathway during reprogramming of human somatic cells has not been described previously and forms the main focus of this manuscript. We report herein that transduction of human fibroblasts with OSKM (both as single and polycistronic Sendai based viruses) induces the activation of JNK/SAPK signaling during the initiation and maturation stage of reprogramming. Downregulation of JNK/SAPK with a specific chemical inhibitor or by RNA interference (RNAi) leads to the emergence of only partially reprogrammed colonies which disaggregate and are lost during the maturation stage of reprogramming. Our data suggest that JNK/SAPK signaling plays an important role in several key processes that are shown to be important during cellular reprogramming namely the induction of mesenchymal to epithelial transition (MET), activation of cellular proliferation as well the maintenance of the pluripotent phenotype. Hence lack of hiPSC colonies and loss of partially reprogrammed cells can be attributed to one or more of these three cellular processes which are tightly regulated by JNK/SAPK signaling.

## Materials and Methods

### hiPSC Generation

Cyto Tune.1‐iPS reprogramming kit (A13780‐01, Invitrogen) and Cyto Tune‐iPS 2.0 Sendai reprogramming kit (A16517, Invitrogen:https://www.thermofisher.com/order/catalog/product/A16517) were used for hiPSC derivation as described in a recent publication by our group 18. CytoTune‐EmGFP Sendai Fluorescence Reporter (A16519, Invitrogen:https://www.thermofisher.com/order/catalog/product/A16519) was used as control.

### Cell Culture

Human neonatal and adult dermal fibroblasts were purchased from Lonza and were cultured in 6 or 12 well tissue culture plates (Iwaki ) in Iscove's Modified Dulbecco's Medium, 10% fetal calf serum (PAA), 2 mM l‐glutamine (PAA), 1% nonessential amino acids (PAA), 100 units/ml penicillin (PAA) at 37°C and 5% CO_2_. Human ESC (H9) and hiPSC lines were cultured under feeder free conditions using (Matrigel: [BD Biosciences], Oxford, UK http://www.bd.com/uk/) as coating substrate (BD Biosciences) and mTESR1 media (Stem Cell Technologies, Cambridge , UK, http://www.stemcell.com/).

### RNA Interference

Stable downregulation of *MKK4, MKK7 and JNK1* in human neonatal and adult fibroblasts was carried out using lentiviral based MISSION shRNAs (*MKK7*: TRCN0000315390 MAP2K7 MISSION shRNA; *MKK4*: TRCN0000039914 MAP2K4 MISSION shRNA and *JNK1*: TRCN0000342626 MAPK8) according to the manufacturer's protocol (Sigma). Briefly, exponentially growing fibroblasts cultures (70%–80% confluent) were transduced with lentiviral particles (MOI = 2; 1.6 × 10^4^ cells). Puromycin selection (5 mg/ml) was used to select stable clones. Replacement of media with fresh puromycin containing media was performed every 2‐3 days until resistant colonies were identified.

### Statistical Analysis

*t* test analysis was used to assess differences between control and RNAi groups. The results were considered significant if *p* < .05. For additional details on materials and methods, please refer to Supporting Information Annex.

## Results

### JNK/SAPKs Kinases are Activated During the Course of Reprogramming

**Figure 1:**
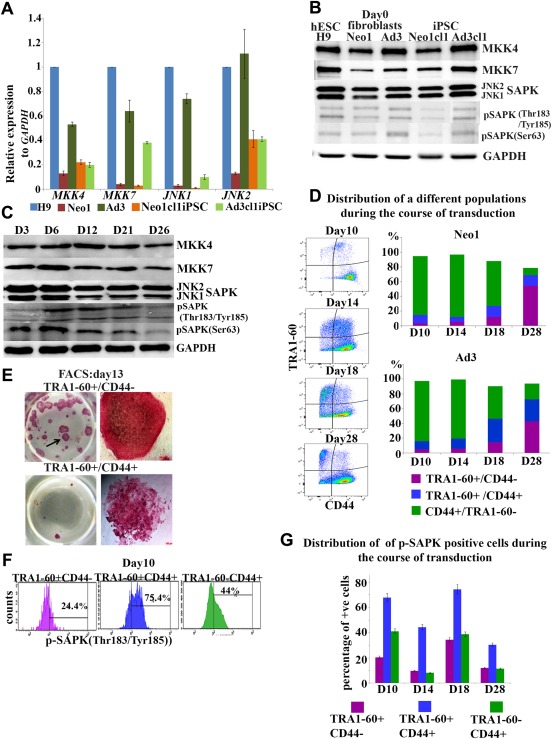
JNK/SAPK signaling is activated during the initiation and maturation stage of reprogramming. **(A)**: Real‐time PCR analysis of *MKK4, MKK7, JNK1* and *JNK2* expression in H9 (p36), neonatal human fibroblasts (Neo1), adult human fibroblasts (Ad3) and human induced pluripotent stem cell (hiPSC) generated therefrom (Neo1cl1iPSC and Ad3cl1iPSC, respectively). Data represent relative expression to *GAPDH* and normalized against H9. Data are presented as mean ± SEM. **(B)**: Western blot analysis showing expression of MKK4, MKK7, JNK/SAPKs, pSAPK(Thr183/Tyr185) and pSAPK (Ser63) in hESC (H9), human neonatal fibroblasts (Neo1), human adult fibroblasts (Ad3) at Day 0 and hiPSC derived therefrom (Neo1cl1iPSC and Ad3cl1iPSC, respectively). **(C)**: Western blot analysis of protein expression of MKK4, MKK7, JNK/SAPKs, pSAPK(Thr183/Tyr185) and pSAPK (Ser63) during the reprogramming of Neo1 fibroblasts. Days of transduction are indicated as D3—Day 3 and so on, correspondently. GAPDH served as loading control. Images are representative of at least three independent experiments. **(D)**: Flow cytometric analysis of the distribution of TRA1‐60+/CD44‐, TRA1‐60+/CD44 + and TRA1‐60‐/CD44 + populations during time course of reprogramming of neonatal (Neo1) and adult fibroblasts (Ad3). This is a representative example of at least three independent experiments. **(E)**: Neo1 fibroblasts undergoing reprogramming were sorted in all four different subpopulation by FACS at day 13 of reprogramming and replated. The resulting colonies were stained by alkaline phosphatase at day 28. TRA1‐60 + /CD44– cells formed numerous AP + colonies (upper panel), while TRA1‐60+/CD44 + cells (lower panel) generated partly reprogrammed colonies. **(F)**: Representative examples of flow cytometric analysis showing the distribution of pSAPK + cells among TRA1‐60+/CD44‐, TRA1‐60+/CD44 + and TRA1‐60‐/CD44 + populations at day 10 of reprogramming of Neo1 fibroblasts. **(G)**: Graphic representation of the percentage of p‐SAPK + cells at different cells populations (TRA1‐60+/CD44‐, TRA1‐60+/CD44 + and TRA1‐60‐/CD44+) during the reprogramming of Neo1 fibroblasts assessed by flow cytometric analysis. Data are presented as mean ± SEM. Abbreviations: FACS, Fluorescence‐activated cell sorting; hESC, human embryonic stem cell; iPSC, induced pluripotent stem cell; JNK, c‐Jun N‐terminal kinase; MKK, MAP kinase kinases; SAPK, stress‐activated protein kinase.

To understand the role of JNK/SAPKs during the generation of hiPSCs we assessed the expression of *JNK1* and *JNK2* and their upstream activators *MKK4* and *MKK7* in two different primary dermal skin fibroblasts (Neonatal/Neo1 and Adult/Ad3), several hiPSCs clones derived therefrom (Fig. 1A, 1B, Supporting Information Fig. 1B) and hESCs (H9). Human ESCs are characterised by high levels of JNK/SAPK activity which has been shown to be important for maintenance of the pluripotent stem cell state 19. In accordance with this, we found the highest levels of mRNA *JNK1* expression in hESCs when compared to several hiPSCs clones derived from two adult fibroblast samples (Fig. 1A, 1B, Supporting Information Fig. 1B); however these differences were not maintained at the protein level across the iPSC clones examined (Fig. 1B). We also observed that neonatal fibroblasts had lower expression of all four kinases examined when compared to adult fibroblasts (Fig. 1A, 1B). These differences were in part maintained in the respective hiPSC lines with the adult derived hiPSC clones showing higher expression of JNK1 when compared to neonatal derived hiPSCs at both transcript and protein level (Fig. 1A, 1B, Supporting Information Fig. 1A, 1B).

Transduction of OSKM caused a significant increase in JNK1 expression in adult fibroblasts and a dual increase in JNK1 and JNK2 expression in neonatal fibroblasts as early as day 3 of reprogramming (Fig. 1A‐1C, Supporting Information Fig. 1A). This was followed by an increase in expression of pSAPK [Tyr 185/Thr 183SAPK]) from day 6 to day 21 in neonatal fibroblasts (Fig. 1B, 1C) and from day 12 to day 21 in adult fibroblasts (Fig. 1B, Supporting Information Fig. 1A). The expression of pSAPK (Ser63) was increased as early as day 3 continuing till day 21 of reprogramming in both neonatal and adult fibroblasts (Fig. 1B, 1C, Supporting Information Fig. 1A). Together these data suggest an increased JNK/SAPK activity during the initiation and maturation stage of reprogramming.

To determine how the four transcription factors (OSKM) individually contribute to JNK/SAPK activation during reprogramming, we performed transduction with each single factor in neonatal fibroblasts and substituted the rest of the factors with an equivalent number of control‐GFP virus particles (Supporting Information Fig. 1C). Transduction with *OCT4, KLF4* and *c‐MYC* contributed mostly to an increase in JNK2 expression, while introduction of *SOX2* increased both JNK1 and JNK2 expression with a preference for JNK1. Transduction with control viral particles alone did not lead to increased JNK1/JNK2 expression or their phosphorylated form (data not shown), indicating that activation of JNK/SAPK pathway during reprogramming is not related to the transduction event, but specifically to introduction of OSKM in somatic cells.

To further confirm the increase in p‐JNK/SAPK expression at a cellular level, we used flow cytometric analysis as described previously 20. This enabled us to follow three cellular subpopulations during the course of reprogramming; fully reprogrammed cells (TRA‐1‐60+CD44‐), partially reprogrammed cells (TRA‐1‐60+CD44+) and fibroblasts (TRA‐1‐60‐CD44+; Fig. 1D, 1E). pSAPK was expressed in all three subpopulations (Fig. 1F, 1G). It is interesting to note that the partially reprogrammed cells showed the highest percentage of pSAPK + expressing cells; however this declined toward the end of the reprogramming period (Fig. 1G). Furthermore, the percentage of pSAPK expressing cells increased in the fully reprogrammed subpopulation from day 14 to day 18 (Fig. 1G) then decreased by day 28, corroborating the Western blotting analysis (Fig. 1B, 1C, Supporting Information Fig. 1A). We obtained the same profile of emergence of the three cellular subpopulations and the same trend of pSAPK activation (Supporting Information Fig. 2A, 2B) upon application of a polycistronic Sendai vector (Cytotune 2.0), demonstrating that activation of JNK/SAPK is independent of hiPSC transduction protocol. Together these data suggest an important role for the activity of JNK/SAPK in fully reprogrammed cells during the maturation stage of reprogramming.

### Inhibition of JNK/SAPK Activation with Chemical Inhibitors Causes Disaggregation and Loss of hiPSC Colonies During Maturation Stage

**Figure 2:**
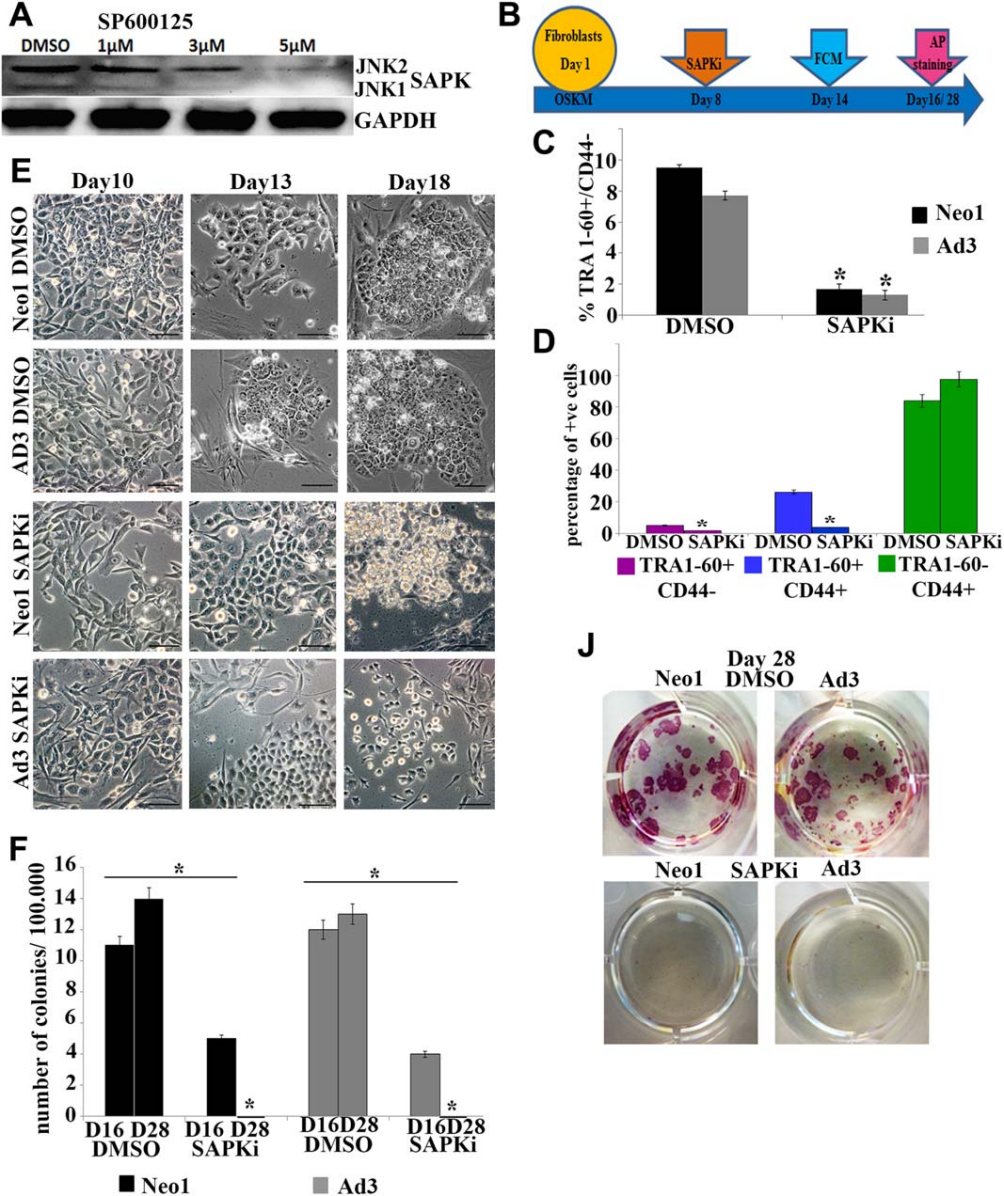
Application of JNK/SAPKs inhibitor (SP60015) abrogates human induced pluripotent stem cell generation. **(A)**: Western blot analysis of JNK/SAPKs downregulation by SP60015 (SAPKi) in hESCs (H9). GAPDH used as a loading control. Images are representative of at least three independent experiments. **(B)**: Schematic representation of inhibitor application (SAPKi) at day 8 during the reprogramming process. **(C)**: Graphic representation of flow cytometric analysis data (day 13) indicating a significant impact of SAPKi application (applied at day 8 for 24 hours) on the percentage of TRA1‐60+/CD44‐ cells. Results are presented as mean ± SEM (*n* = 3). **(D)**: Graphic representation of flow cytometric analysis data (day 13) demonstrating a significant impact of SAPKi application on TRA1‐60+/CD44‐ and TRA1‐60+/CD44 + subpopulations generated during reprogramming of Neo1 fibroblasts. Results are presented as mean ± SEM (*n* = 3). **(E)**: Phase–contrast observation showing the morphology of partially reprogrammed colonies arising during the reprogramming of Neo1 and Ad3 fibroblasts treated with DMSO or SAPKi for 24 hours at day 8, scale bars = 100 µm. **(F)**: Graphic representation of total colony numbers at day 16 and 28 of reprogramming in SAPKi and DMSO treated Neo1 and Ad3 fibroblasts. Data are presented as mean ± SEM (*n* = 3). **(J)**: Alkaline phosphatase staining at day 28 confirmed the absence of true AP + colonies from neonatal and adult fibroblasts undergoing reprogramming and treated with SAPKi at day 8 of transduction for 24 hours. (C, D, F): *, *p* < .05. Abbreviations: AP, alkaline phosphatase; DMSO, dimethyl sulfoxide; FCM, flow cytometric and morphological analysis; JNK, c‐Jun N‐terminal kinase; SAPK, stress‐activated protein kinase.

To test the function of JNK/SAPKs in generation of hiPSCs we used a small molecule SP600125, which has been shown to significantly inhibit expression of all three *JNK* genes namely *JNK1, JNK2*, and *JNK3* 21. Our data show that 5 µM SP600125 (named SAPKi thereafter) is sufficient to downregulate the expression of JNK1 and JNK2 in hESCs (Fig. 2A) corroborating previous data published in mouse ESCs 22. To investigate the impact of JNK/SAPK inhibition in the reprogramming process, we used SAPKi for 24 hours at different time points as summarized in Figure 2B and Supporting Information Figure 3. Our results indicate that application of SAPKi had a detrimental effect on hiPSC generation regardless of the time of application, for no hiPSC colonies were obtained at the end of the transduction period from either adult or neonatal fibroblasts (Fig. 2F‐2J, Supporting Information Fig. 3). In all cases, flow cytometric analysis indicated a significant decrease in the percentage of emerging hiPSCs (TRA‐1‐60+CD44‐; Fig. 2B‐2D). Furthermore, SAPKi application affected the TRA1‐60 + populations specifically (partially reprogrammed and fully reprogrammed distinguished by presence or absence of CD44 respectively, Fig. 2C, 2D) as no reduction in the TRA‐1‐60‐CD44 + population of dermal skin fibroblasts was observed (Fig. 2D). In control cultures (treated with DMSO vehicle only) we observed morphological changes (cells rounding up, starting to group together and showing morphology typical of pluripotent stem cells) which led to the emergence of hiPSC colonies with clear compact edges as early as day 16 (Fig. 2E, Supporting Information Fig. 3). In SAPKi treated cultures, we observed formation of colonies with morphology typical of partially reprogrammed cells; however most of these started to disintegrate as early as day 16 (Fig. 2E, 2D) and by day 18 all these partially reprogrammed colonies were lost from the culture (Fig. 2E).Thus, formation of hiPSCs colonies showing morphological features of pluripotent stem cells was not observed in the presence of SAPKi. Assessment of total colony number at the mid‐point (day 16) as well as hiPSC colonies at day 28 (identified by alkaline phosphatase staining) corroborated the morphological and flow cytometric analysis and indicated no viable hiPSC colonies upon application of SAPKi (Fig. 2F, 2J), suggesting that JNK/SAPK activity is important for generation of hiPSCs colonies.

### *MKK4, MKK7* and *JNK1*/*SAPK1* are Indispensable for hiPSC Generation

**Figure 3:**
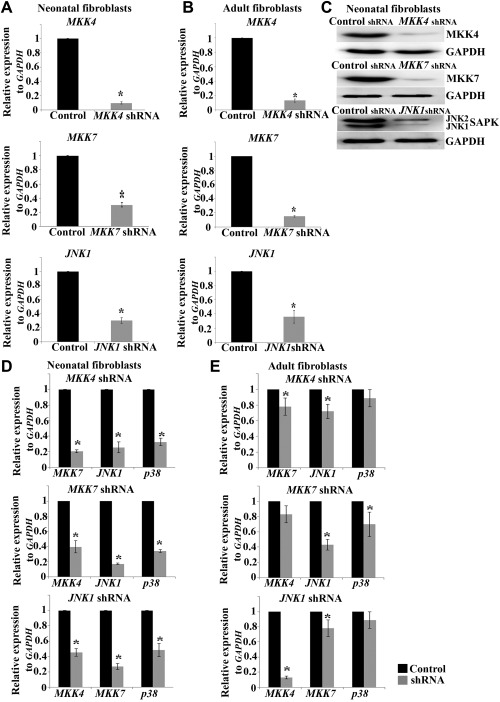
Assessment of *MKK4, MKK7* and *JNK1* downregulation in neonatal **(A)** and adult **(B)** fibroblasts by real‐time quantitative RT‐PCR analysis. Data represent relative expression to *GAPDH* and normalized against Control shRNA. Results are presented as average ± SEM (*n* = 3). *t* test analysis was carried out to assess differences in gene expression between the control and shRNA groups. Western blot analysis of MKK4, MKK7 and SAPK expression in Control shRNA and *MKK4* shRNA, *MKK7* shRNA and *JNK1* shRNA neonatal cells **(C)**. Analysis of the crosstalk between upstream and downstream MKK4/MKK7‐JNK1 signaling pathways in neonatal **(D)** and adult fibroblasts **(E)** revealed by real‐time quantitative RT‐PCR analysis. Results are presented as average ± SEM (*n* = 3). *, *p* < .05. Abbreviations: JNK, c‐Jun N‐terminal kinase; MKK, MAP kinase kinases; SAPK, stress‐activated protein kinase; shRNA, short hairpin RNA.

Application of chemical inhibitors can induce transient downregulation of JNK/SAPK which may mask some of their effects on cellular reprogramming. To investigate if permanent downregulation of JNK/SAPK yields the same or more extensive impact we generated stable cell lines of neonatal and adult fibroblasts in which *MKK4, MKK7* and *JNK1/SAPK1* (*JNK1*) were downregulated by shRNA. Successful downregulation of each of the three genes was confirmed by qRT‐RCR at the mRNA level (Fig. 3A, 3B) and protein level (Fig. 3C). We observed that downregulation of *MKK4, MKK7* and *JNK1* resulted in a more effective repression of downstream targets in neonatal fibroblasts when compared to their adult counterparts (Fig. 3D, 3E). In part this may be explained by higher expression of these kinases in adult fibroblasts (Fig. 1A, 1B) and possibly by the existence of additional compensatory mechanisms.

Morphological observation of *MKK4, MKK7* and *JNK1* shRNA treated fibroblasts revealed differences between neonatal and adult cells (Supporting Information Fig. 4A, 4B). Neonatal fibroblasts continued to proliferate very slowly after passage 3, thus corroborating previously published literature reporting reduced proliferation and G2/M phase arrest in mouse embryonic fibroblasts 16. We also observed that *MKK4, MKK7* and *JNK1* shRNA adult fibroblasts stopped growing after the first passage and demonstrated spindle shape and flattened morphology similar to senescent cells 23. Despite this, we were unable to detect activation of senescence markers (β‐gal, p15, p16, p19) in either neonatal or adult fibroblasts deficient in JNK signaling (data not shown).

**Figure 4:**
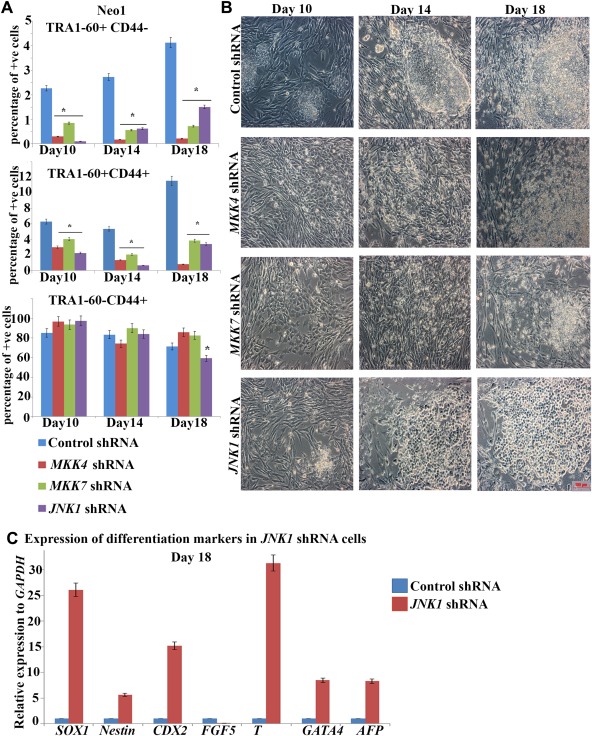
Inhibition of MKK4, MKK7 and JNK1 dependent signaling is detrimental for human induced pluripotent stem cells generation. **(A)**: Graphic representation of flow cytometric analysis on the different cellular subpopulations during reprogramming of *MKK4, MKK7* and *JNK1* deficient Neo1 fibroblasts. Results are presented as mean ± SEM (*n* = 3). *, *p* < .05. **(B)**: Phase‐contrast images of representative colonies arising during reprogramming of the control, *MKK4*‐ *MKK7* and *JNK1* deficient Neo1 fibroblasts. Scale bar = 100 µm. **(C)**: Real‐time quantitative RT‐PCR analysis for ectoderm, mesoderm and endoderm markers at day 18 in *JNK1* shRNA transduced cells. Data represent relative expression to *GAPDH* and normalized against Control shRNA. Results are presented as average ± SEM (*n* = 3). Abbreviations: JNK, c‐Jun N‐terminal kinase; MKK, MAP kinase kinases; SAPK, stress‐activated protein kinase; shRNA, short hairpin RNA.

We performed transduction of neonatal and adult control and *MKK4, MKK7, JNK1* shRNA fibroblasts with OSKM and as in the case of SAPKi we observed greatly reduced numbers of emerging hiPSCs (TRA1‐60+CD44‐) and partially reprogrammed cells (TRA‐1‐60+CD44+; Fig. 4A, 4B). Quantitative RT‐PCR analysis at 7 days post transduction indicated that downregulation of *MKK4, MKK7* and *JNK1* shRNA was maintained (Supporting Information Fig. 5). On a morphological level, partially reprogrammed cells were detected from neonatal fibroblasts transduced with *JNK1, MKK4* and *MKK7* shRNA as early as day 14 (Fig. 4B); however these started to disaggregate by day 18, corroborating the data observed with SAPKi. Transduced adult fibroblasts exhibited a few minor morphological changes during the first 10 days of reprogramming; however they did not form either partially reprogrammed or fully reprogrammed colonies (data not shown). The same profile of colony disaggregation and a significant reduction in the percentage of true emerging hiPSC (TRA1‐60+CD44‐) was observed when *JNK1, MKK4* and *MKK7* shRNA neonatal fibroblasts were reprogrammed with a Sendai polycistronic vector (Cytotune 2.0; Supporting Information Fig. 6). To understand whether the absence of a true hiPSCs from *JNK1* shRNA transduced cells was associated with cellular differentiation we performed qRT‐PCR analysis of the expression of three germ layer differentiation markers on cells collected from remaining colonies at day 18. This analysis indicated the upregulation of al differentiation markers examines apart from *FGF5*, suggesting that the onset of differentiation can be an additional reason for the absence of hiPSC from *JNK1* shRNA cells (Fig. 4C). In accordance with the above results, no alkaline phosphatase positive hiPSC colonies were obtained from either neonatal or adult fibroblasts, indicating that MMK4, MKK7 and JNK1 are indispensable for successful reprogramming.

### Impact of JNK/SAPK Downregulation on Pluripotent Stem Cells

To understand the deleterious effect of JNK/SAPK downregulation during the reprogramming process, we inhibited JNK/SAPK pathway in hESC via administration of SAPKi. Human ESCs have been characterized by high JNK activity 24 and inhibition of this pathway with chemical inhibitors has been shown to result in cellular differentiation, decreased OCT4 and NANOG expression 19. Consistent with published results, we observed loss of typical pluripotent stem cell morphology (Supporting Information Fig. 7A) downregulation of *OCT4* expression (Supporting Information Fig. 7B) and an increase in *KLF4* expression, corroborating previously published data on mESCs 17. Also, in agreement with previous reports 17 we found increased expression of differentiation markers characteristic for neuroectoderm (*SOX1, NESTIN*), trophoectoderm (*CDX2*) and primitive endoderm (*AFP*; Supporting Information Fig. 7C). No change in cell survival was observed as a result of SAPKi application (data not shown); however an increase in G1 phase at the expense of S phase was observed upon cell cycle analysis by flow cytometry (Supporting Information Fig. 7D). Together these data suggest that JNK activity is required for the maintenance of pluripotency; hence its downregulation during the reprogramming process can provide one of the underlying factors for the emergence of colonies with partially reprogrammed phenotype and lack of fully reprogrammed hiPSCs.

A recent report has suggested that heat shock treatment of hESC results in activation of SAPK/JNK signaling and reduced expression of OCT4 due to increased binding of heat shock factor 1 to the *OCT4* promoter 25. Although this finding may appear contradictory to our data presented above, we would like to point out that the difference may be due to physiological stimuli. While hESC data presented in this work were collected from normal culture conditions (37°C and normoxic oxygen levels), the Buyn et al. findings relate to the “heat shock” conditions which represent stress conditions and are likely to affect many other signaling pathways in hESC in addition to the JNK/SAPK pathway.

### Impact of JNK/SAPK Downregulation on Cellular Proliferation and Cell Cycle Regulation in Somatic Cells

To better understand the changes induced in neonatal and adult fibroblasts by downregulation of the JNK/SAPK pathway, we administered SAPKi to culture media. Similarly to our observations obtained with *JNK1* shRNA (Supporting Information Fig. 4), we observed that SAPKi treated fibroblasts became more elongated, flattened and in some cases spindle shaped (Supporting Information Fig. 7E). Growth curve analysis also indicated a slower rate of proliferation in SAPKi treated fibroblasts (Supporting Information Fig. 7F) supporting previously published data 26. This was further confirmed by cell cycle analysis which demonstrated that downregulation of JNK/SAPKs resulted in cell accumulation in G1 phase at the expense of S phase cells (Supporting Information Fig. 7G). Western blot analysis also indicated the downregulation of the downstream target, c‐Jun known to be important for cellular proliferation (Supporting Information Fig. 7H).

**Figure 5:**
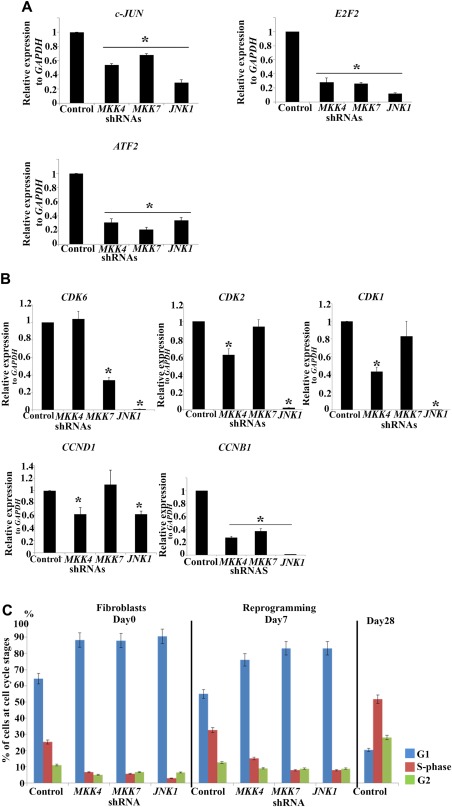
*MKK4, MKK7* and *JNK1* downregulation results in changes in the expression of key genes involved in cellular proliferation and cell cycle progression. **(A, B)**: Real‐time quantitative PCR analysis of *c‐JUN, E2F2, ATF2* (A) and *CDK6, CDK2, CDK1, CCND1 and CCNB1* (B) in Control shRNA (Control) and *MKK4, MKK7* and *JNK1* deficient Neo1 fibroblasts at Day 1 of reprogramming. Results are presented as mean ± SEM (*n* = 3). *, *p* < .05. **(C)**: Flow cytometric cell cycle analysis of *MKK4, MKK7* and *JNK1* shRNA treated fibroblasts during the reprogramming process. Results are presented as mean ± SEM (*n* = 3). Abbreviations: JNK, c‐Jun N‐terminal kinase; MKK, MAP kinase kinases; shRNA, short hairpin RNA.

To understand the molecular changes underpinning JNK/SAPK inhibition, we performed quantitative RT‐PCR analysis of JNK target genes involved in cellular proliferation, namely *c‐Jun, E2F2* and *ATF2*. Since the effect of SAPKi may be transient, we performed this analysis on neonatal and adult fibroblasts treated with *MKK4, MKK7* and *JNK1* shRNA described in Figure 3A and 3B. We found that *c‐JUN, ATF2* and *E2F2* were significantly downregulated in all cases (Fig. 5A). Of interest are also the findings that key cyclin dependent kinases (*CDK6, CDK2 and CDK1*) were downregulated in at least one of the shRNA treated samples. Furthermore *CCNB1*, a key cyclin involved in both G1/S and G2/M regulation was downregulated in *MKK4, MKK7* and *JNK1* shRNA treated samples, while Cyclin D1 (*CCND1*) was downregulated in *MKK4* and *JNK1* shRNA cells (Fig. 5B). Flow cytometry cell cycle analysis revealed that in contrast to fibroblasts treated with control shRNA, which demonstrated a characteristic somatic cell cycle distribution at day 0, *MKK4*, *MKK7 and JNK1*shRNA treated fibroblasts showed an increased cell accumulation at G1 phase at the expense of S and G2/M phase prior to reprogramming (day 0) and at day 7 of reprogramming (Fig. 5C). Together these data suggest an impaired cell cycle regulation and proliferation as a result of JNK/SAPK downregulation.

### Importance of JNK/SAPK Signaling for the MET

**Figure 6:**
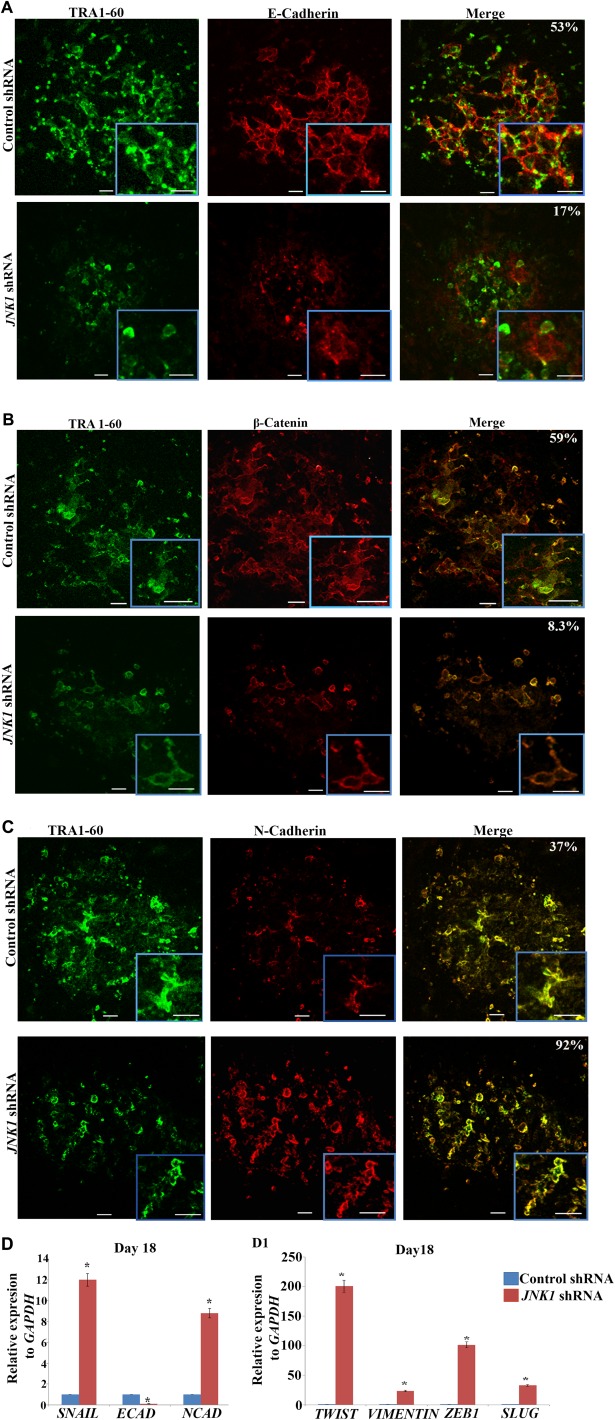
Downregulation of *JNK1* expression results in disruption of the E‐cadherin/β‐Catenin network and increased N‐Cadherin junctional staining in emerging partially reprogrammed colonies. Double‐immunofluorescence staining of transduced control and *JNK1* deficient Neo1 fibroblast for E‐cadherin **(A)**, β‐Catenin **(B)** and N‐cadherin **(C)** together with TRA1‐60 (green) on day 18 post transduction with OCT3/4, SOX2, KLF4, and c‐MYC assessed by confocal microscope. Scale bars represent 50 µm. Images are representative of at least three independent experiments. The percentage of double‐positive colonies is shown on the top right hand corner of the merged images. **(D, D1)**: Real‐time quantitative PCR analysis of *SNAIL, ECAD, NCAD* and *TWIST, VIMENTIN, ZEB1* and *SLUG* at day 18 of reprogramming. Data represent relative expression to *GAPDH* and normalized against Control shRNA. Results are presented as mean ± SEM (*n* = 3). *, *p* < .05. Abbreviations: JNK, c‐Jun N‐terminal kinase; shRNA, short hairpin RNA.

MET is an important process during somatic cell reprogramming and is orchestrated by the suppression of pro‐epithelial to mesenchymal transition (EMT) signals and activation of an epithelial signature in reprogrammed cells 27. During this process, two of the key reprogramming factors SOX2 and OCT4 suppress the EMT mediator SNAIL, while KLF4 induces epithelial gene expression including E‐cadherin 27. Ultrastructural visualization of this process has shown that until day 6 of reprogramming, human fibroblasts retain their mesenchymal characteristics. The MET occurs between days 6 and 12 and this is followed by maturation of the epithelial phenotype from day 12 to day 18 28. Although traditionally JNKs have been thought to be activated in response to apoptotic, proliferation and stress signals, more recently it has been shown that they can also act as cell junction regulators 29 and can be involved in cell migration which is tightly linked to dynamic formation as well as dissolution of cell‐cell junctions 29, 30. Given the importance of MET in hiPSC formation we asked whether inhibition of JNK/SAPK signaling leads to loss of E‐cadherin expression‐dependent adhesion, followed by loss of cell‐to‐cell communication resulting in disintegration of partially reprogrammed colonies and their loss from culture. Immunocytochemical analysis of OSKM treated control fibroblasts indicated formation of a bright network of E‐cadherin and β‐catenin plasma membrane staining in TRA‐1‐60 positive cells (Fig. 6A, 6B), indicating formation of adherent junctions between the cells. In contrast, E‐cadherin and β‐catenin staining was observed in very few of the cells present in the partially reprogrammed TRA‐1‐60 positive colonies obtained from reprogramming of *JNK1* shRNA treated fibroblasts (Fig. 6A, 6B). Furthermore, the bright E‐cadherin and β‐catenin networks which ensure the cell‐cell adhesion and communication were missing in reprogrammed *JNK1* shRNA treated fibroblasts. Assessment of N‐cadherin expression, a marker of fibroblasts that fail to undergo MET and further convert into hiPSC 31 indicated a higher number of expressing cells in *JNK* deficient cells (Fig. 6C), further supporting the disruption of MET during the reprogramming of JNK deficient samples. Quantitative RT‐PCR analysis indicated downregulation of epithelial marker (*E‐cadherin*) and upregulation of mesenchymal markers (*SNAIL, N‐cadherin, TWIST, VIMENTIN, ZEB1* and *SLUG*) under both Cytotune 1 and 2 Sendai reprogramming (Fig. 6D, Supporting Information Fig. 8), further supporting the disruption of MET during the reprogramming of JNK deficient samples.

## Discussion

In this manuscript, we investigated the expression of key components of JNK/SAPK signaling and found that this pathway is activated as early as day 3 of reprogramming with both partially reprogrammed cells (TRA‐1‐60+CD44+) and fully reprogrammed cell populations (TRA‐1‐60+CD44‐) showing an increase in the number of pSAPK expressing cells until day 18, regardless of the reprogramming protocol used. In contrary to our first expectations, we found that inhibition of JNK/SAPK signaling pathway either by chemical inhibitors or RNAi mediated downregulation of *MKK4, MKK7* or *JNK1* resulted in the complete abrogation of hiPSC colony formation and emergence of partially reprogrammed cells which were lost during the maturation stage of reprogramming. Together these data highlight the importance of this signaling pathway in human somatic cell induced reprogramming which to the best of our knowledge has not been reported previously. In contrast to our findings, it has been reported that *Jnk1*‐/‐ and *Jnk2*‐/‐ murine fibroblasts exhibit a greater potency for reprogramming due to increased Klf4 activity which is phosphorylated by Jnk1 and Jnk2 through inhibitory phosphorylation at Thr residues 224 and 225 17. These differences in the role of JNK signaling during the reprogramming of mouse versus human somatic cells can reflect different modes of actions for this pathway in each species; however this needs to be investigated further. We were also surprised to see the lack of functional redundancy between various components of key signaling molecules involved in JNK signaling as downregulation of *MKK4, MKK7* and *JNK1* in fibroblasts led in all cases to complete absence of hiPSC colonies. Notwithstanding this, we would like to point out that all single component downregulation by RNAi led to further changes in the expression of additional components of this pathway (e.g., *MKK4* shRNA resulted in downregulation of *MKK7* and *JNK1*) and/or parallel pathways such as p38, thus outlining the complexity of regulation of this pathway which has been reported previously and must be taken into account when investigating the function of separate signaling components 32, 33, 34. Furthermore, we observed some differences in the expression of key components of JNK/SAPK signaling between neonatal and adult fibroblasts as well as their response to JNK/SAPK inhibition which indicates differences in the mode of operation of this pathway in these two cell types. Nevertheless, the activation window of JNK/SAPK signaling during the reprogramming process and the outcome of JNK/SAPK downregulation in the reprogramming of both cell types were very similar, suggesting that at least the JNK/SAPK functions related to reprogramming are likely to be conserved in both cell types. Human cord blood and hair follicle epithelial cells have emerged as new sources of somatic cells for reprogramming because of their accessibility. Recent reports suggest that JNK/SAPK signaling is required in cord blood cells to prevent the onset of senescence 35, while regulating apoptosis in hair follicle epithelial cells 36. While our experiments have only been performed in dermal skin neonatal and adult fibroblasts thus preventing to make sound conclusions about the applicability of our findings in other cell types, the well‐known role of JNK/SAPK signaling in maintaining cell survival and proliferation may suggest a critical role for this pathway during the reprogramming process; however this has to be further investigated experimentally.

The functions of JNK/SAPK signaling are complex and encompass a wide range of cellular processes which yield different outcomes in specific cell types 37. Given the increased expression of key components of JNK/SAPK signaling during the initiation and maturation phases of reprogramming, we sought to identify its involvement in several processes shown to be important during this time window namely induction of MET 38, silencing of tumor suppressor and cell cycle inhibitor genes 8, induction of apoptosis 39 and induction of cell proliferation 40, 41. Consistent with previous reports 42, we found that inhibition of JNK signaling did not have any impact on the apoptosis of fibroblasts undergoing reprogramming (data not shown); hence the link between apoptosis induction and JNK signaling can be excluded. JNK signaling has been shown to be required for the expression of c‐Jun and JunD, two essential components of the AP1 transcription factor which is essential for cell proliferation and prevention of senescence often associated with impaired JNK signaling 43. Despite repeated investigations, we did not observe expression of senescence associated β‐galactosidase in fibroblasts with deficient JNK signaling achieved through chemical inhibition or RNAi. Neither did we see an increase in the expression of cell cycle inhibitors including p15, p16, p19 which have been linked to onset of senescence in various cell types 44, 45. These findings could reflect differences in JNK outcomes in vivo versus in vitro experiments (tissue culture) which have been reported previously, or the time lag that is needed from cell cycle arrest to detectable expression of these markers. Despite lack of senescence marker expression, we observed a significant decrease in cellular proliferation, increased percentage of cells in G1 phase of the cell cycle at the expense of S phase and reduced expression of key cell cycle components involved in G1/S transition (such as *CDK6, CDK2, CDK1, CCDN1*) and S phase progression and G2/M transition (e.g., *CCNB1*). It is important to note that such expression changes were specific for each component of the JNK signaling pathway downregulated; however in all cases simultaneous downregulation of at least one CDK and one partnering Cyclin, an event that has been shown to be important for efficient reprogramming 40 was observed. These findings were further corroborated by decreased expression of cellular proliferation markers *c‐Jun, E2F2* and *ATF2* in all three shRNA treated samples consistent with previous reports indicating an important role for MKK4, MKK7 and JNK1 for cell cycle progression 16, 46. High cellular proliferation rate akin to ESC state is an important early event for cellular reprogramming 40. This leads us to suggest that suppressed cellular proliferation and cell accumulation at the G1 phase (both known as reprogramming barriers; 47) at the onset of reprogramming of *JNK1, MKK4* and *MKK7* deficient neonatal and adult fibroblasts is a key reason for the complete lack of hiPSC observed during reprogramming of these samples.

The efficiency of reprogramming can be promoted by the onset of epithelial expression markers concomitantly with the repression of mesenchymal markers in cells undergoing reprogramming during the 6th until the 12th day of reprogramming, known as initiation phase 48, 49. This is precisely the window during which the JNK signaling is activated; hence we asked the question of whether MET transition is affected in JNK deficient cells. Our data suggest that while control cells showed well organized TRA‐1‐60 + colonies characterized by plasma membrane staining of E‐cadherin and β‐catenin indicating formation of adherent junctions typical of epithelial cells, JNK deficient cells displayed a reduced percentage of cells expressing these markers at both cellular and mRNA levels, which suggests that fewer cells are able to undergo MET transition, thus leading to inefficient reprogramming. Furthermore, the cellular networks that guarantee cell‐cell communication while present and well organized in control cells were lacking in JNK/SAPK deficient fibroblasts. It has been suggested that expression of E‐cadherin and the presence of intact adherent junctions are essential for maintenance of pluripotency in hESC by ensuring access to critical autocrine signals and by promoting cell‐cell exchange of signals through the gap junctions 49, 50. In this context, it is interesting to note that all colonies that emerged during reprogramming of JNK/SAPK deficient fibroblasts were partially reprogrammed and completely disaggregated and subsequently lost during the maturation stage of reprogramming thus leading us to suggest that deficient JNK/SAPK signaling followed by impaired cell to cell contact is likely to be one of the causative factor that counts for the partially reprogrammed phenotype and for the loss of colonies.

Our inhibition experiments were performed during the initiation stage of reprogramming. In all cases despite the time of JNK/SAPK downregulation we obtained no hiPSC colonies. However, we did not perform JNK/SAPK downregulation studies at the stabilization stage where hiPSC colonies are present and continue to grow and develop. There were two main reasons behind this decision: (1) JNK/SAPK activation occurs during the initiation and maturation stage and shows a drop during the stabilization stage and (2) our data presented in this manuscript together with published reports indicate loss of pluripotency in hESCs upon JNK downregulation 19, which would suggest that downregulation of JNK/SAPK signaling after the emergence of hiPSC colonies would result in their differentiation and thus provide a further push toward loss of true bona fide hiPSC colonies.

## Conclusion

**Figure 7:**
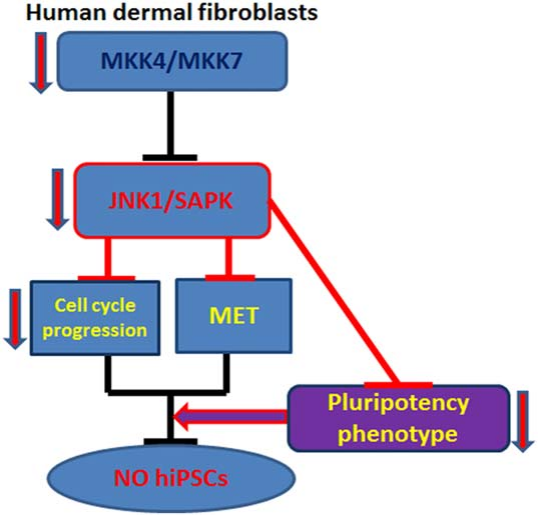
Schematic summary showing the impacts of MKK4, MKK7 and JNK1 signaling on hiPSC generation. Abbreviations: hiPSC, human induced pluripotent stem cell; JNK, c‐Jun N‐terminal kinase; MET, mesenchymal to epithelial transition; MKK, MAP kinase kinases; SAPK, stress‐activated protein kinase.

Together our data indicate an increase in JNK/SAPK activity during the initiation and maturation phases of reprogramming regardless of the reprogramming protocol and an indispensable role for the generation of hiPSC colonies. Furthermore, we have shown that inhibition of JNK/SAPK signaling results in reduced cell proliferation, disruption of MET and loss of pluripotent phenotype which either singly or in combination prevent establishment of pluripotent colonies as shown in the summary (Fig. 7).

## Author Contributions

I.N.: designed and performed research, analyzed data and contributed to paper writing, final approval of manuscript; E.S., J.M., R.A., J.P.: performed some research and data analysis, final approval of manuscript; V.C.: performed some research and data analysis, final approval of manuscript; G.A.: performed some research, final approval of manuscript; D.J.E.: contributed to design of study and paper writing, final approval of manuscript; L.A.: designed research, contributed to paper writing, final approval of manuscript and fund raising; M.L.: designed research, data analysis, wrote the paper, final approval of manuscript and fund raising.

## Disclosure of Potential Conflicts of Interest

The authors indicate no potential conflicts of interest.

## Supporting information

Supplementary Information

Supplementary Information

Supplementary Information

Supplementary Information

Supplementary Information

Supplementary Information

Supplementary Information

Supplementary Information

Supplementary Information

Supplementary Information
