# Supplementary figures and images for: JNK/SAPK Signaling Is Essential for Efficient Reprogramming of Human Fibroblasts to Induced Pluripotent Stem Cells
Source: Stem Cells. 2016 Mar 4;34(5):1198–212. doi: 10.1002/stem.2327 (PMC4982072; doi:10.1002/stem.2327)

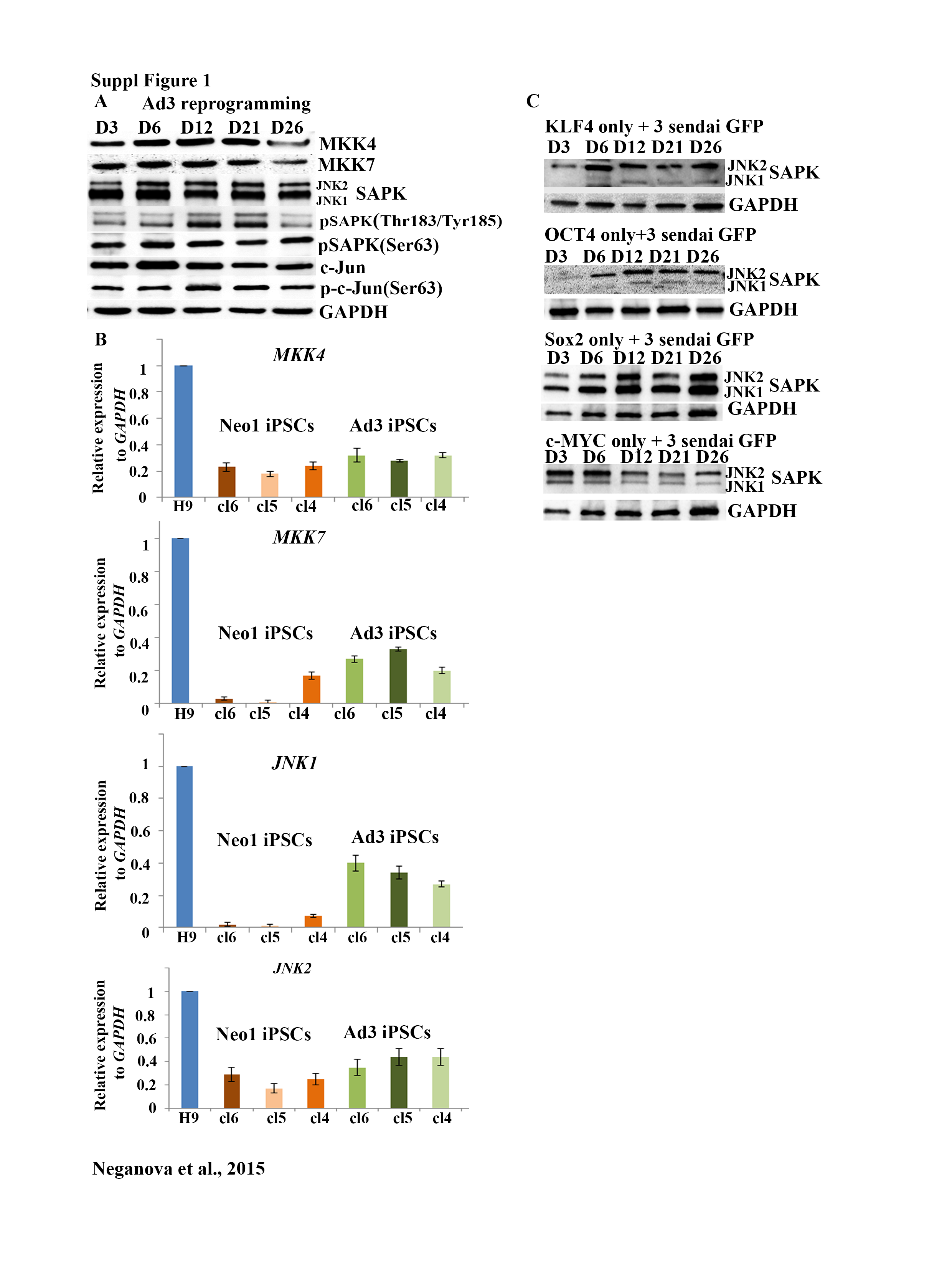

Supplement: Supplementary file 1 — Supplementary Information [file STEM-34-1198-s001.tif]

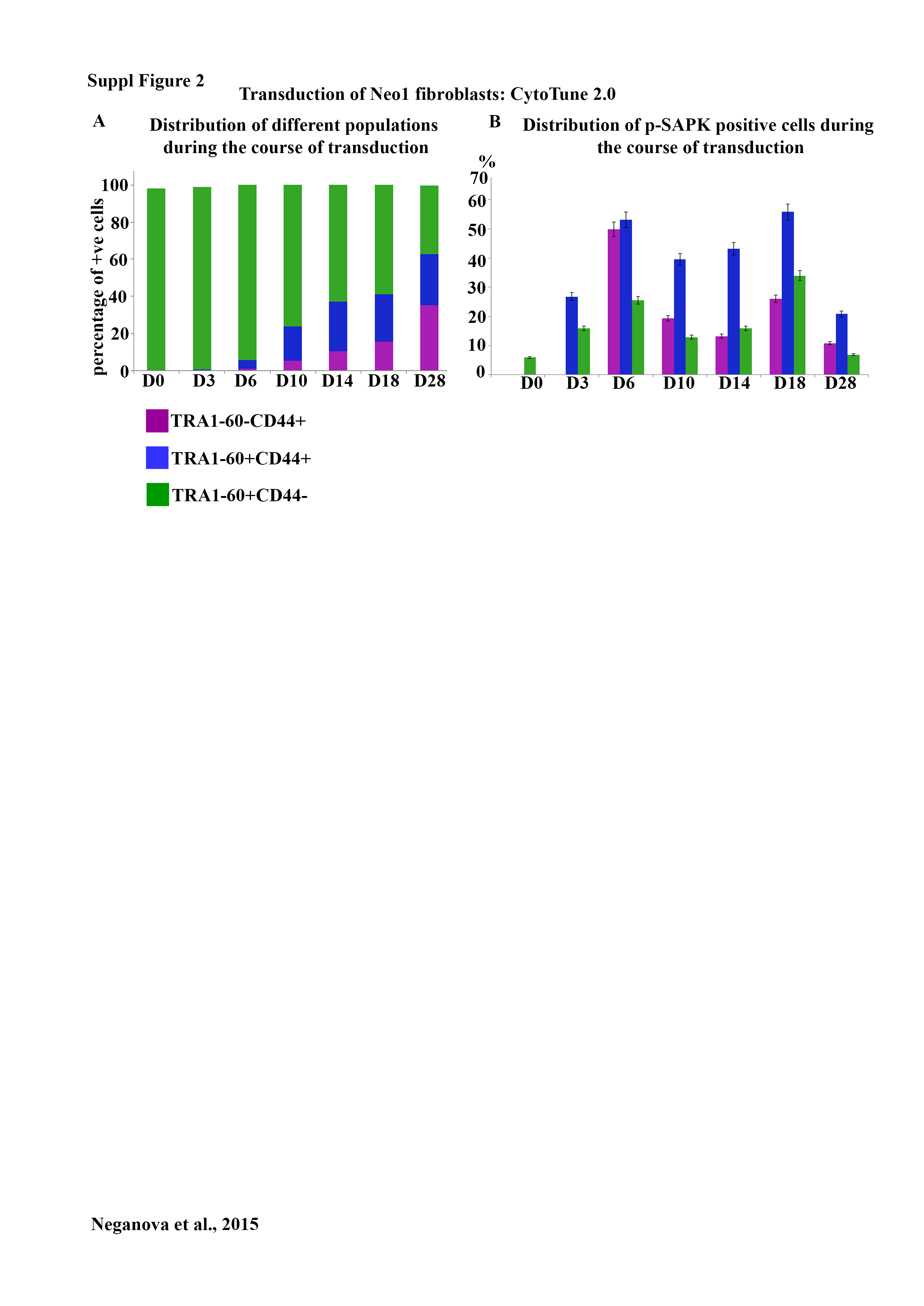

Supplement: Supplementary file 2 — Supplementary Information [file STEM-34-1198-s002.tif]

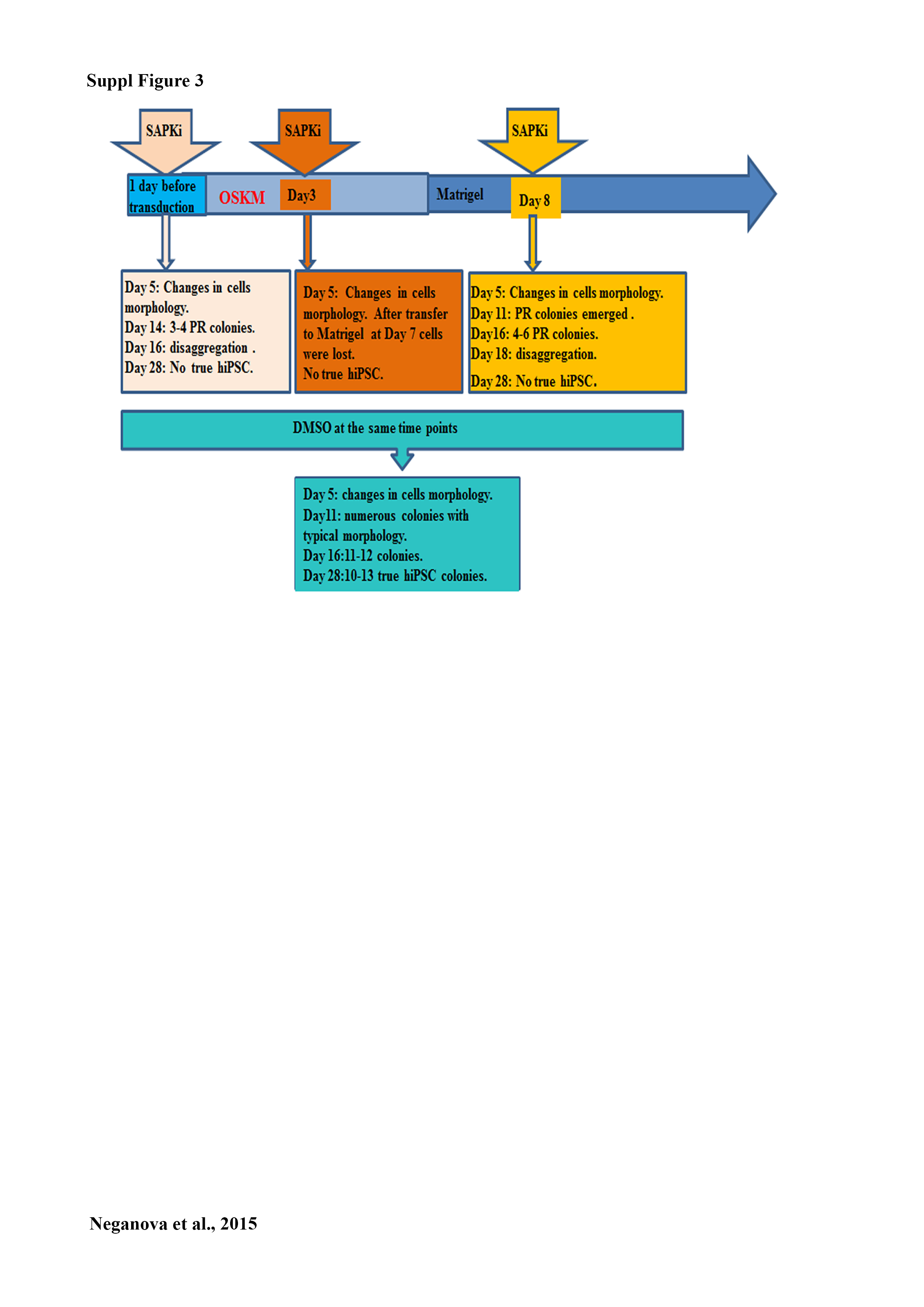

Supplement: Supplementary file 3 — Supplementary Information [file STEM-34-1198-s003.tif]

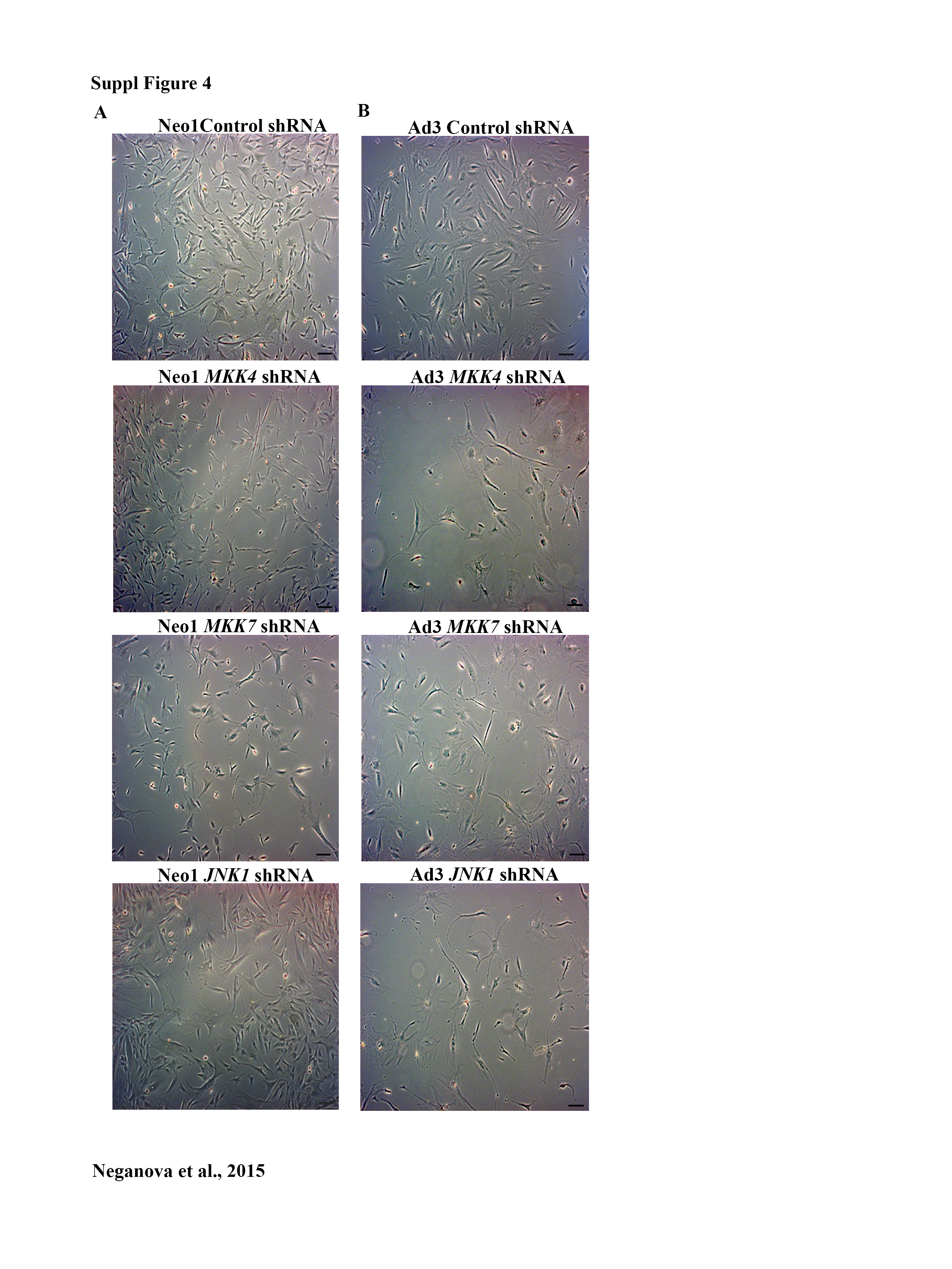

Supplement: Supplementary file 4 — Supplementary Information [file STEM-34-1198-s004.tif]

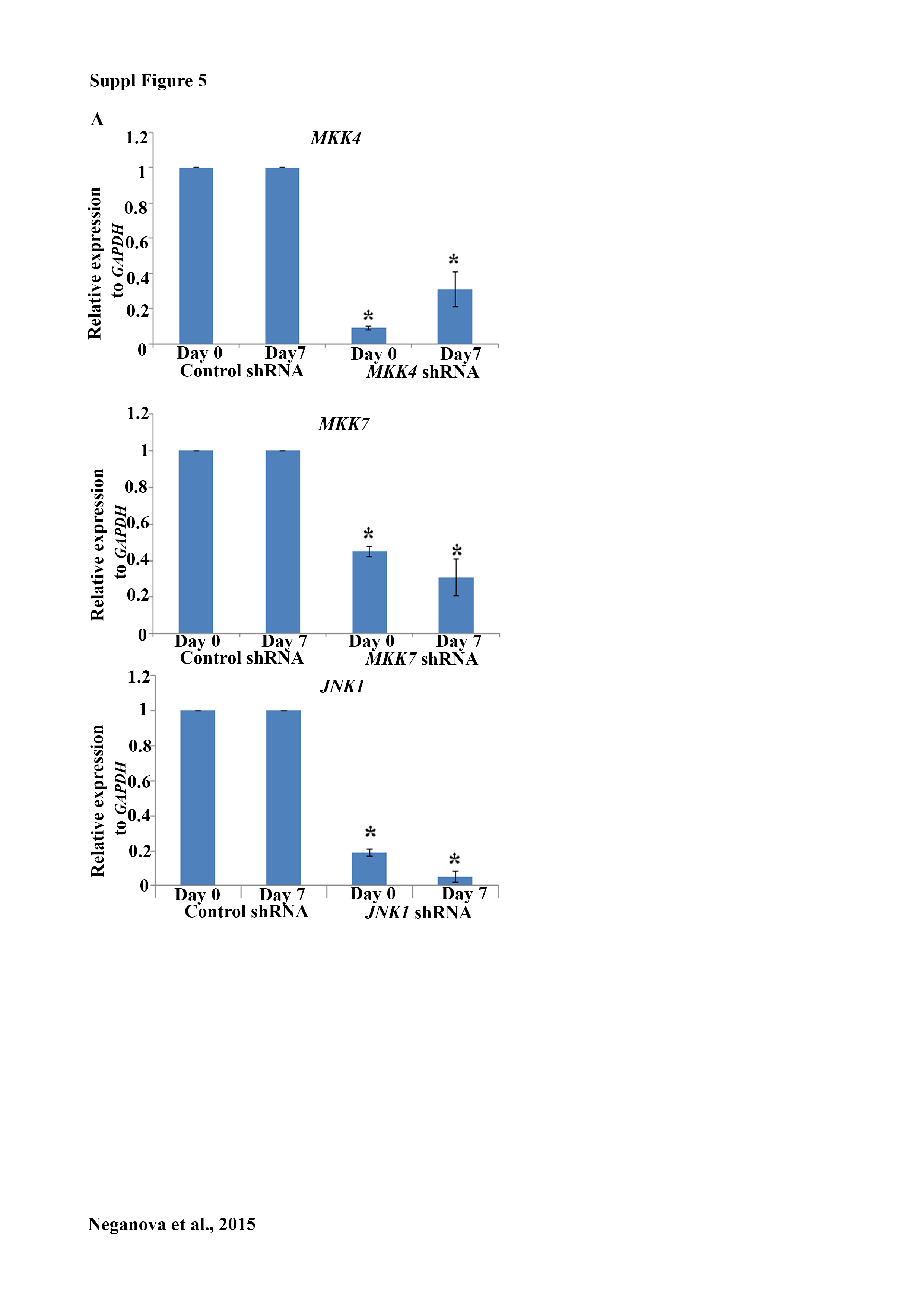

Supplement: Supplementary file 5 — Supplementary Information [file STEM-34-1198-s005.tif]

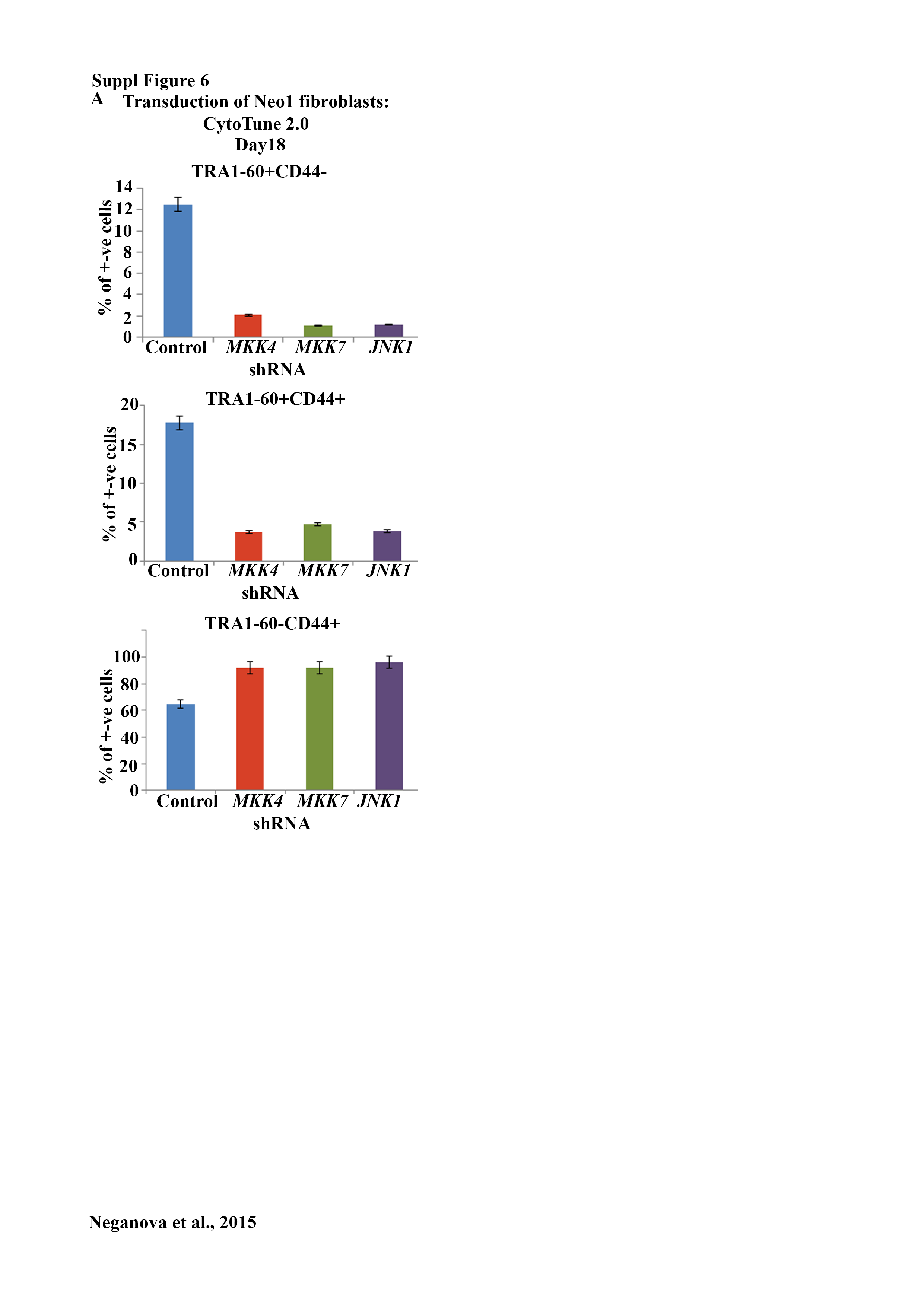

Supplement: Supplementary file 6 — Supplementary Information [file STEM-34-1198-s006.tif]

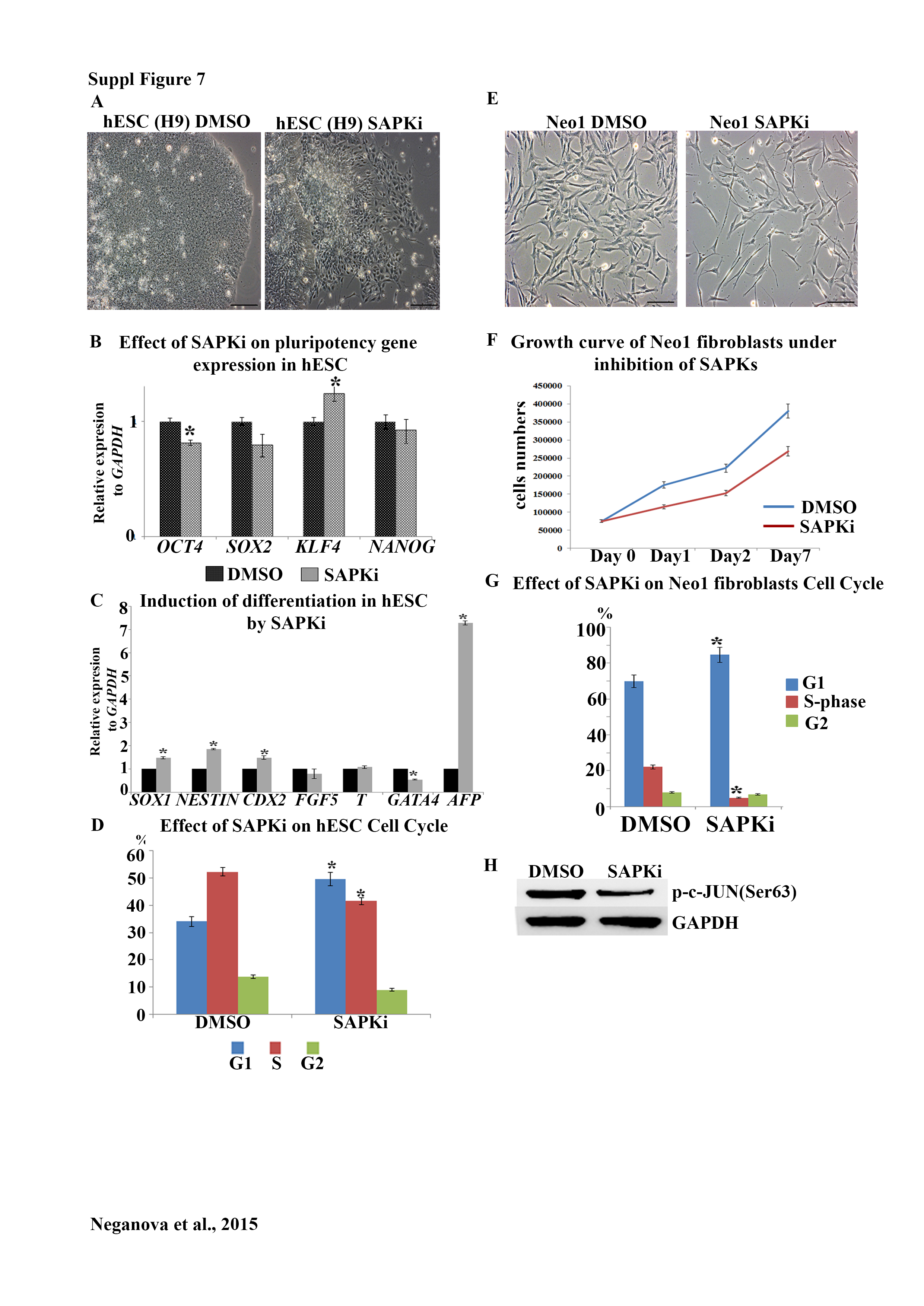

Supplement: Supplementary file 7 — Supplementary Information [file STEM-34-1198-s007.tif]

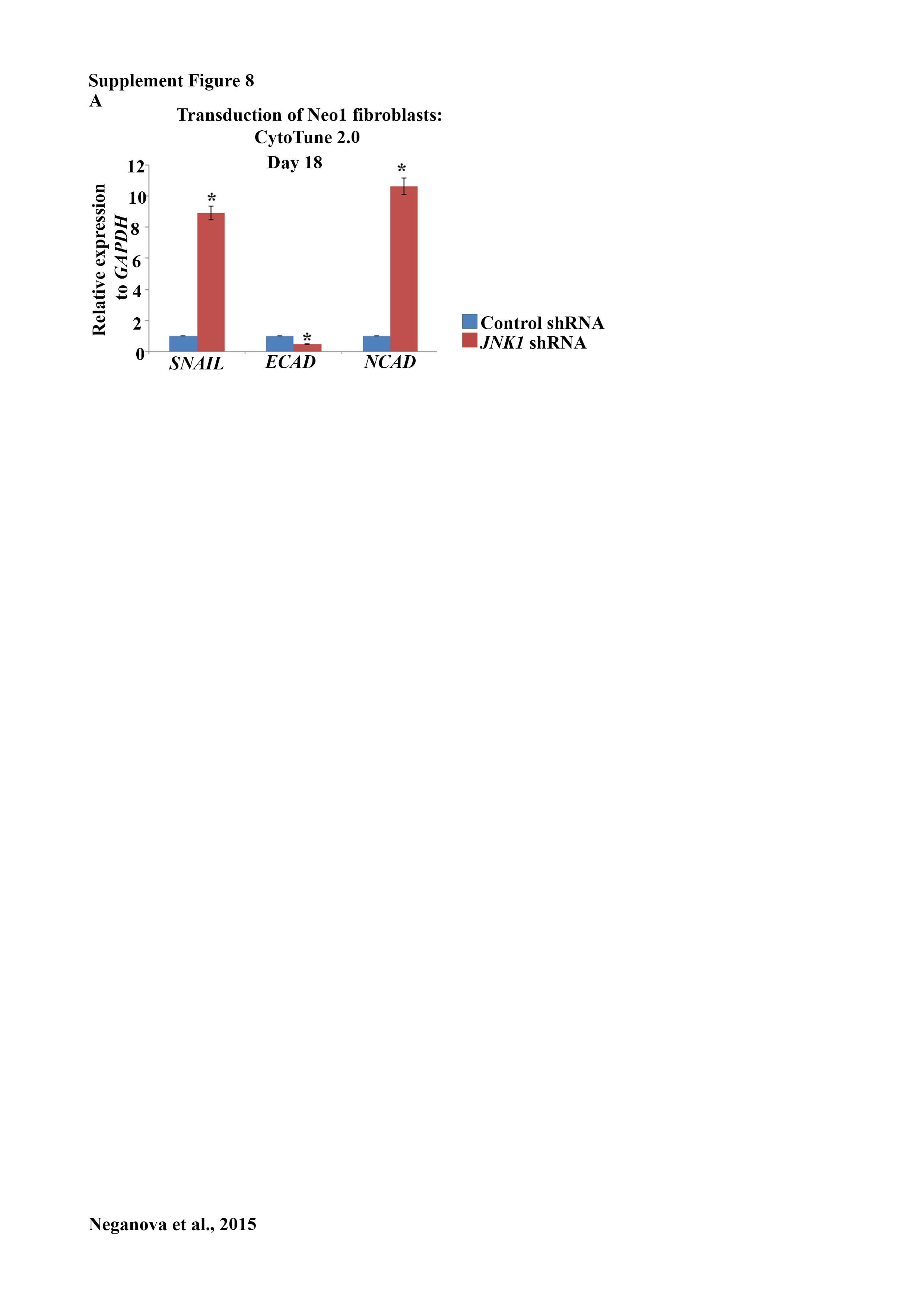

Supplement: Supplementary file 8 — Supplementary Information [file STEM-34-1198-s008.tif]
