# Supplementary material for: JNK/SAPK Signaling Is Essential for Efficient Reprogramming of Human Fibroblasts to Induced Pluripotent Stem Cells
Source: Stem Cells. 2016 Mar 4;34(5):1198–212. doi: 10.1002/stem.2327 (PMC4982072; doi:10.1002/stem.2327)
Supplement: Supplementary file 9 — Supplementary Information [file STEM-34-1198-s009.docx]

**Suppl Table 1**

| **Gene** | **Forward primer sequence (5’→3’)** | **Reverse primer sequence (5’→3’)** |
| --- | --- | --- |
| *A-CATENIN* | GCAACTAAACAGGAAGGGATG | GATGACGAAGAGCACAGATGG |
| *AFP* | CTTTGGGCTGCTCGCTATGA | ATGGCTTGGAAAGTTCGGGTC |
| *ATF2* | GCCAATTGT//CCCTGTACCA | GTCCTAACCAATCCGCTACC |
| *c-JUN* | GCTCTGGGAAGTGAGTTCG | CTCCCGCACTCTTACTTGTC |
| *CCNB1* | AACTTTCGCCTGAGCCTATTTT | TTGGTCTGACTGCTTGCTCTT |
| *CCND1* | GGCGGAGGAGAACAAACAG | AGGCGGTAGTAGGACAGGA |
| *CDK1* | TTTTCAGAGCTTTGGGCACT | CCATTTTGCCAGAAATTCGT |
| *CDK2* | TGTACCTCCCCTGGATGAAG | CATCCTGGAAGAAAGGGTGA |
| *CDK6* | AGGATAAGCCAACCTGAGAC | ACTGAGAGTATGACTGGCAA |
| *CDX2* | CTCGGCAGCCAAGTGAAAAC | CTCCTTTGCTCTGCGGTTCT |
| *E-CADHERIN* | TGCCCAGAAAATGAAAAAGG | GTGTATGTGGCAATGCGTTC |
| *E2F2* | AGGAGCTGATGAACACGGA | CGGCAATCACTGTCTGCT |
| *FGF5* | ATTTGCTGTGTCTCAGGGGAT | CTGTGAACTTGGCACTTGCAT |
| *FOXA2* | GCATTCCCAATCTTGACACGGTGA | GCCCTTGCAGCCAGAATACACATT |
| *GAPDH* | TGCACCACCAACTGCTTAGC | GGCATGGACTGTGGTCATGAG |
| *GATA4* | TCCAAACCAGAAAACGGAAG | AAGGCTCTCACTGCCTGAAG |
| *JNK2* | TCACATGGAGCTGGATCATGAAA | AAGTTAGTGCACGCTGTCCG |
| *MKK4* | ACTTCGGCATCAGTGGACAG | CTCCAGACATCAGAGCGGAC |
| *MKK7* | ACGTTCATCACCAACACGGA | GGTAGTACAGCGCCTTCACA |
| *N-CADHERIN* | ACAGTGGCCACCTACAAAGG | CCGAGATGGGGTTGATAATG |
| *NESTIN* | GAGAGGGAGGACAAAGTCCC | GAGAGGGAGGACAAAGTCCC |
| *p38* | GGGTTACGTGTGGCAGTGAA | ACGTCCAACAGACCAATCACA |
| *SLUG* | GGGGAGAAGCCTTTTTCTTG | TCCTCATGTTTGTGCAGGAG |
| *SNAIL* | CCTCCCTGTCAGATGAGGAC | CCAGGCTGAGGTATTCCTTG |
| *SAPK1* | AGCACCTTCATTCTGCTGGAA | AGCGAGTCACTACATAAGGCG |
| *SOX1* | GGAATGGGAGGACAGGATTT | ACTTTTATTTCTCGGCCCGT |
| *T* | CAGTGGCAGTCTCAGGTTAAGAAGGA | CAGTGGCAGTCTCAGGTTAAGAAGGA |
| *TWIST* | CCTCCCTGTCAGATGAGGAC | TCTGGAGGACCTGGTAGAGG |
| *VIMENTIN* | GAGAACTTTGCCGTTGAAGC | GCTTCCTGTAGGTGGCAATC |
| *ZEB1* | CCACACGACCACAGATACGG | CCTGAGGAGAACTGGTTGCC |
