# Supplementary material for: JNK/SAPK Signaling Is Essential for Efficient Reprogramming of Human Fibroblasts to Induced Pluripotent Stem Cells
Source: Stem Cells. 2016 Mar 4;34(5):1198–212. doi: 10.1002/stem.2327 (PMC4982072; doi:10.1002/stem.2327)
Supplement: Supplementary file 10 — Supplementary Information [file STEM-34-1198-s010.docx]

**Western immunoblotting**

Protein extraction, western blotting and antibody/antigen complex detection were performed as published previously (47)**.** Primary antibodies included: anti- MKK4 (5C10); anti- MKK7 (4172); anti-SAPK (9252); anti –pSAPK (Thr183/Tyr185) (9251); anti-c-JUN (60A8); anti –pc-JUN (Ser63) 54B3 (2361) all from Cell Signalling. The antibody to GAPDH (Abcam, ab9485) was used as a loading control.

**Immunocytochemistry and confocal microscopy**

Cells were washed three times with phosphate-buffered saline (PBS), prior to being fixed with 4% paraformaldehyde for 10 minutes and subsequently permeabilised with 0.25% Triton X-100 in PBS for 30 minutes at room temperature. Unspecific binding was blocked by incubation of samples in PBS containing 10% normal goat serum (Invitrogen) and 1% bovine serum albumin (Sigma) for 30 minutes. Cells were incubated with primary antibodies overnight at 4°C and secondary antibodies for 1 hour at room temperature. Primary antibodies used in this study were anti-Tra-1-60 FITC conjugate (Merck Millipore), anti-E-Cadherin (Cell Signaling Technology), anti-β-Catenin and anti N-Cadherin (both BD Biosciences). Cells were covered by a cover slip using Vectashield (Vector Laboratories).

The images were acquired with a Nikon A1R laser scanning confocal microscope (Nikon, http:// nikon.com) using a CFl Plan Apochromat VC 20×/0.75 objective. The excitation wavelength was as follows: 488 nm for fluorescein isothiocyanate (FITC), 561nm for Cy3. The emission aperture for fluorescence detection was 500–550 nm for FITC and 570–620 nm for Cy3. Two-dimensional images were acquired with a size of 1,024 × 1,024 pixels. All confocal images were acquired through sequential scan mode and using the same settings. The distribution of fluorescence was analyzed using the NIS-Elements Confocal Software. Quantification was performed by counting Tra-1-60-positive cell clusters per well of a 6-well plate per experiment.

**Quantitative RT-PCR**

Cells were harvested and total RNA extracted using TRIzol (Invitrogen, 15596-026), according to manufacturer’s instructions. RNA was treated with DNase 1 (Ambion) and cDNA was generated by reverse transcription of 100ng of total RNA using the Superscript VILO cDNA synthesis kit (Invitrogen, 11754-050). Quantitative PCR was performed on triplicate biological cDNA repeats using SYBR® Green PCR Master Mix (Invitrogen, 4309155) and the QuantStudio 7 system (Life Technologies). Samples were normalised using *GAPDH* and *PBGD*. All primer sequences are listed in **Suppl. Table 1**.

.

**Flow cytometric analysis**

Cells were disassociated using Versene (EDTA) (Lonza), washed with PBS and fixed in paraformaldehyde (PFA; 2% final concentration in PBS) at 37°C for 10min. After washing with PBS, the cells were permeabilised with pre-chilled methanol (-18°C) and incubated at 4 °C for 30 minutes, followed by a washing step. 0.2-0.5 x 10^6^ cells were resuspended in a total volume of 200 µl PBS containing 1% BSA and incubated with appropriate amounts of anti-CD44-BV421 (Cat.N.562890, BD Biosciences; 1:300 dilution), anti-TRA-1-60-FITC (Cat.N. FCMAB115F, Merck Millipore; 1:100 dilution) and Phospho-SAPK/JNK-Alexa Fluor 647 (Cat.N. 9257, Cell Signaling Technology; 1:50 dilution; Mouse mAb Ig1 Isotype Control Alexa Fluor 647 Conjugate Cat.N.4843, Cell Signaling ) monoclonal antibodies for 1 hour on a shaker plate in the dark at room temperature. Finally, samples were washed using BD FACS™ Lyse Wash Assistant (BD Biosciences) and immediately analysed on a flow cytometer.

FACS analysis was performed using BD FACS Canto II flow cytometer with FACSDiva software (BD Biosciences). A minimum of 20,000 events were recorded for each sample. Fluorescence minus One control (for each antibody) was used to gate the subpopulations.

**Alkaline Phosphatase detection** was performed with Alkaline Phosphotase detection kit (SCR004, Millipore) according to manufacture instructions.
